# Spatial Distribution of Trypanosomes in Cattle From Western Kenya

**DOI:** 10.3389/fvets.2020.00554

**Published:** 2020-08-28

**Authors:** Velma Kivali, Alice N. Kiyong'a, Jenna Fyfe, Philip Toye, Eric M. Fèvre, Elizabeth A. J. Cook

**Affiliations:** ^1^International Livestock Research Institute, Nairobi, Kenya; ^2^Biomedical Sciences, Edinburgh Medical School, University of Edinburgh, Edinburgh, United Kingdom; ^3^Institute of Infection, Veterinary and Ecological Sciences, University of Liverpool, Liverpool, United Kingdom

**Keywords:** trypanosomiasis, tsetse, cattle, Kenya, spatial distribution

## Abstract

African Animal Trypanosomiasis (AAT) is a tsetse-transmitted protozoan disease endemic in “the tsetse belt” of Africa. Past studies investigating the epidemiology of the disease rarely focused on spatial distribution when reporting the prevalence. The challenge of understanding the spatial epidemiology of the disease is further confounded by low-sensitive parasitological techniques used in field investigations. This study aimed to identify trypanosome species in cattle and their spatial distribution in western Kenya. Low-sensitive microscopic analysis and highly-sensitive polymerase chain reaction (PCR) techniques were also compared to better understand the epidemiology of *Trypanosoma* infections by use of the geographical information system (GIS). Blood samples from 888 cattle, collected between August 2010 and July 2012, were examined for *Trypanosoma* parasites by light microscopy and PCR. The spatial distribution of *Trypanosoma* positive cases by species were mapped and overlaid on the map for tsetse distribution. The estimated prevalence was 4.17% by PCR compared to 2.48% by microscopy. Trypanosomes were detected in tsetse free areas. *Trypanosoma vivax* and *Trypanosoma b. brucei* were identified, but not the zoonotic *Trypanosoma b. rhodesiense*. This study demonstrated the importance of geospatial data analysis to understand the epidemiology of the parasite, to inform future research and formulate control strategies.

## Introduction

Trypanosomiasis is a disease of humans and animals caused by protozoan parasites of the genus *Trypanasoma* and is transmitted by tsetse flies of the genus *Glossina*. It is endemic in 38 countries in sub-Saharan Africa (1). The disease exists in two forms; Human African Trypanosomiasis (HAT), a neglected tropical disease that is fatal if left untreated (2) and African Animal Trypanosomiasis (AAT). AAT is associated with substantial economic losses to farmers and is a major constraint in the fight against poverty in the affected countries (3). *Trypanasoma* species that affect cattle are *Trypanasoma vivax, Trypanasoma congolense*, and *Trypanasoma brucei*. Trypanosomiasis has a zoonotic aspect whose etiological agents are *Trypanasoma b. rhodesiense* and *Trypanasoma b. gambiense*. Cattle and other domestic animals are the reservoirs for these zoonotic trypanosomes which are transmitted cyclically between humans and animals by tsetse flies (4).

Diagnosis of trypanosomiasis can be clinical, parasitological, serological, or by use of molecular techniques (5). Control of trypanosomiasis is through the combination of both vector control, to reduce the tsetse fly population, and prophylactic chemotherapy (6). Trypanocidal drugs are also used to treat clinical cases of bovine trypanosomiasis (7).

Kenya lies partially within the tsetse belt of sub-Saharan Africa. According to Machila et al. (8) trypanosomiasis is endemic to the western and coastal regions of Kenya. Epidemiological studies of both HAT and AAT have been conducted in different parts of the country (8–11).

Previous studies have shown that AAT is a major constraint to cattle production in western Kenya (10). Epidemiological studies in this area in 2004 estimated a prevalence of 20.1% of trypanosomiasis in cattle, 8.4 and 17.4% in pigs from two different sites in the region and a prevalence of <5% in small ruminants (11). A study conducted in Teso and Suba Districts in western Kenya in 2006 investigated the spatial distribution of AAT and showed the majority of *Trypanasoma* infections were caused by *T. vivax* outside the tsetse belt (12).

This study aimed to identify the different trypanosome species infecting cattle in Busia county and surrounding areas and map their distribution. Additionally, it sought to establish whether human infective *T. brucei*, i.e., *T. b. rhodesiense* was present in cattle in this region.

## Materials and Methods

Cattle samples used in this study were collected between August 2010 and July 2012 as part of a cross-sectional study conducted in Busia region of the former Western province of Kenya (13). Sampling was conducted within a 45 km radius of Busia town spreading into the current Siaya, Kakamega, and Bungoma Counties. The sample size was determined and adjusted using the Survey package in the R program version 3.1.1 (http://cran.r-project.org/) and the study design powered by an estimated lowest prevalence of 5% of disease in cattle with a standard error of 2%. The primary sampling unit was a homestead defined as the place where family members shared an evening meal. Eight hundred and eighty eight cattle samples were collected from 416 randomly selected homesteads. These were apparently healthy animals on farms included in a cross-sectional survey of zoonotic infections. Sampling was done after seeking consent from the head of all eligible homesteads.

Structured questionnaires were administered prior to sampling to capture animal characteristics, such as age, sex, and breed. Venous blood was collected from the jugular vein of each animal into ethylenediaminetetraacetic acid (EDTA) coated tubes which were barcoded and labeled with the animal identity. The samples were transported in upright position to the project laboratory in Busia and stored at −40°C, before being shipped to Nairobi for subsequent analysis. All samples were examined by both microscopy and PCR and the results interpreted in parallel.

Thin and thick blood smears were prepared in the field at the point of collection from the marginal ear veins of the study cattle. Microscopic diagnosis on thick and thin blood smears was used to determine presence or absence of the trypanosomes. Thick smears were examined using X10 objective lens and the number of parasites counted in each of 100 fields. Thin smears were also examined at X10 and then at X100 under oil immersion and number of parasites in every 100 fields was recorded. The results were interpreted as per the OIE recommendations, i.e., one parasite in 10 fields examined was interpreted as low, one parasite in 5 fields was interpreted as medium and more than one parasite in 1 field was interpreted as high.

DNA was extracted from all EDTA blood samples using the MagNa Pure LC DNA Isolation Kit I (Cat. No. 03 003 990 001) and the MagNa Pure LC 2.0 Instrument (Roche Applied Science, Mannheim, Germany). Conventional species specific PCR testing was performed on extracted DNA for detection of *T. b. brucei, T. congolense, T. vivax*. All *T. b. brucei* positive samples were further analyzed to determine if they were zoonotic, i.e., *T. b. rhodesiense*. Previously described PCR protocols (14–16) were used for:

- T. b. brucei

forward primer sequence GAATATTAAACAATGCGCAG

reverse primer CCATTTATTAGCTTTGTTGC,

- T. congolense

forward primer sequence CGAGAACGGGCACTTTGCGA

reverse primer GGACAAACAAATCCCGCACA,

- T. vivax

forward primer sequence CAGCTCGGCGAAGGCCACTTCGCTGGGGTG

reverse primer sequence TCGCTACCACAGTCGCAATCGTCGTCT CAAGG,

- T. b. rhodesiense

forward primer sequence ATAGTGACAAGATGCGTACTCAACGC

reverse primer sequence AATGTGTTCGAGTACTTCGGTCACGCT.

Annealing temperatures for the primers were determined using gradient PCR. Reaction volume for each sample consisted of 1X DreamTaq Green PCR Master Mix (ThermoFisher Scientific; Waltham, Massachusetts), 10 μM of forward and reverse species-specific primers, DNA and nuclease-free water. Thermocycling was done on the C1000 touch Thermo cycler (BIO-RAD, Hercules, California, United States) and amplicons were detected using gel electrophoresis.

Descriptive analysis was performed to determine the distribution of the results obtained based on age, sex, and breed of the animals. This was achieved using the “frequency” tool of IBM SPSS® version 20. A Cohen's Kappa test was run (17) to compare the level of agreement between microscopy and PCR.

ESRI ArcGIS 10.3.1 software was used to spatially map all the positive cases using the x and y coordinates. Additional data layers' describing the tsetse distribution in the study area was obtained from a GIS database available at the International Livestock Research Institute website (http://www.ilri.org/GIS). A positive case was defined as positive using either microscopy, PCR or both diagnostic techniques.

A spatial risk map was generated to identify areas with the greatest relative risk for trypanosome infection. The density of positive cases of trypanosomes was assessed using Kernel smoothing in the *spatstat* package (18) in R. This was done with a fixed bandwidth of 5 km and correlation for edge effects. The kernel intensity of positive homesteads was divided by the kernel intensity of the all homesteads in the study area creating a risk surface.

## Results

Out of the 888 cattle analyzed, 65.3% were female and almost all of them belonged to the shorthorn Zebu and Zebu breeds (Supplementary Table).

The estimated prevalence of trypanosome species detected by both microscopy and PCR was 4.17% (95% CI 3.931–4.409). Thirty four animals were infected with a single trypanosome species while three animals had mixed trypanosome infections of *T. vivax and T. b. brucei. T. congolense* constituted the smallest proportion of all trypanosomes found in cattle in the study site (Table 1).

**Table 1:** Prevalence of trypanosoma species identified based on the test used.

**Trypanosome species (Test used)**	**Positive/sample size**	**Apparent prevalence (95% confidence interval)**
All species tested	37/888	4.17 (3.931–4.409)
*T. brucei (thick smears)*	1/888	0.11 (0.131–0.089)
*T. brucei (thin smears)*	1/888	0.11 (0.089–0.131)
*T. brucei (PCR)*	8/888	0.90 (0.920–0.880)
*T. congolense (thick smear)*	4/888	0.45 (0.483–0.417)
*T. congolense (thin smear)*	2/888	0.23 (0.258–0.202)
*T. congolense savannah (PCR)*	0/888	0.00 (0.000–0.000)
*T. vivax (thick smear)*	13/888	1.46 (1.514–1.406)
*T. vivax (thin smear)*	13/888	1.46 (1.514–1.406)
*T. vivax (PCR)*	12/888	1.35 (0.468–0.332)

### Spatial Distribution and Relative Risk

**Figure 1:**
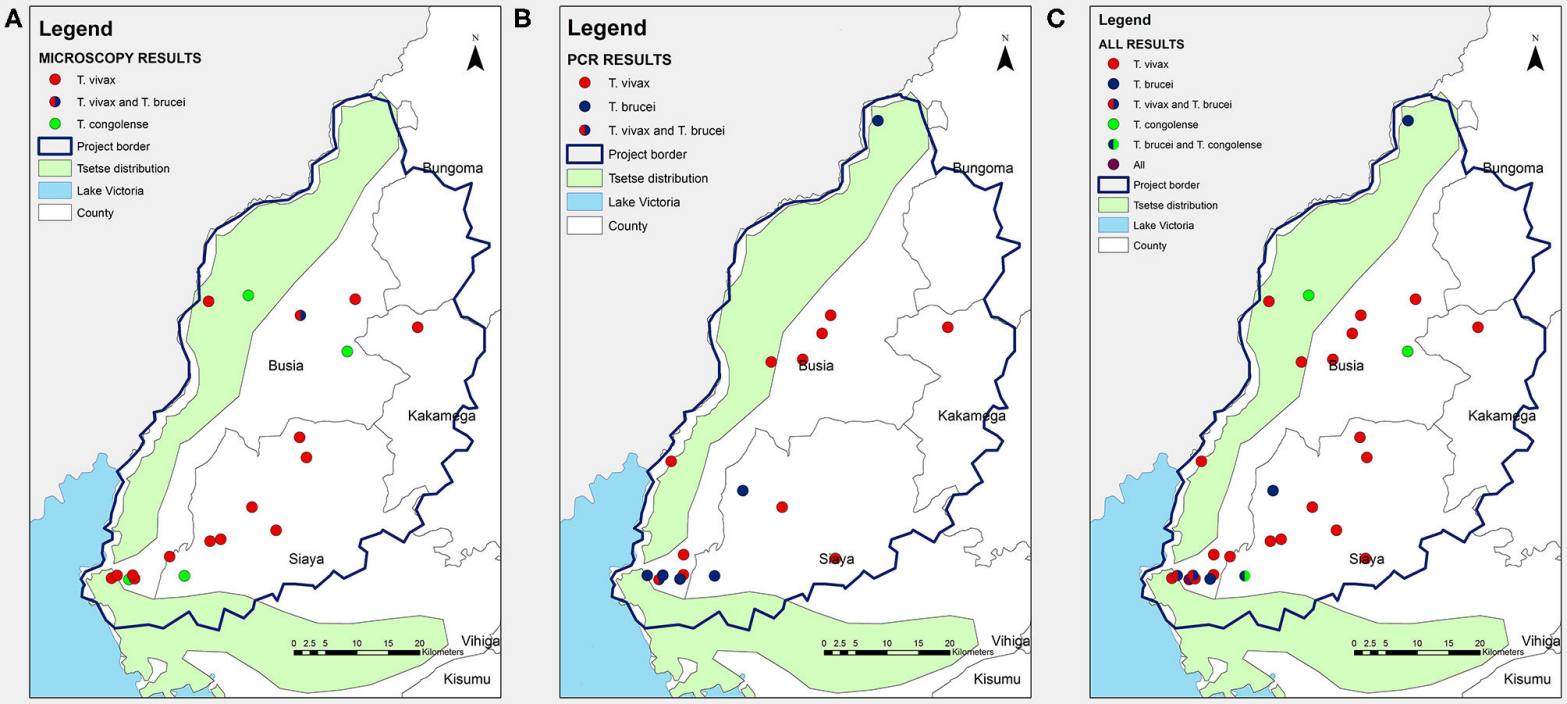
Map of the distribution of homesteads with trypanosome positive cattle in Busia region detected by **(A)** Microscopy, **(B)** PCR, and **(C)** Microscopy and PCR.

The locations of all positive cases were mapped as shown in Figure 1. Most of the infected cattle were located in the western and south-western parts of the study area (Figures 1A–C). The addition of the tsetse fly distribution layer to the map revealed that there were trypanasome positive cattle in tsetse-free areas in the eastern part of the study area. The distribution of positive cattle was the same for both diagnostic tests used (Figures 1A,B).

**Figure 2:**
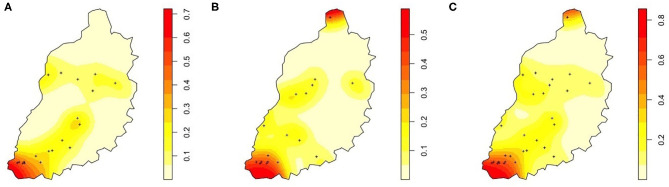
Spatially-smoothed risk map for trypanosomes in cattle in Busia region detected by **(A)** Microscopy, **(B)** PCR, and **(C)** Microscopy and PCR. The points indicate locations of hometeads with positive trypanasome samples. Risk is measured as a ratio between 0 and 1 with results closest to 1 having the highest risk and demonstrated by increasing color intensity.

Spatial relative risk maps (Figure 2) also demonstrated that areas of greatest relative risk for trypanosome infection were the south western parts of the study area around Lake Victoria.

### Comparison of Microscopy and PCR

*T. vivax* was the most common infection detected by microscopy (Figure 1A) and *T. brucei* was the most common infection detected by PCR (Figure 1B). Only *T. vivax* results provided a statistically reasonable number of positives hence possible to determine the level of agreement between the PCR and microscopy results. A Cohen's Kappa test was run and the results revealed a moderate agreement between the two tests, i.e., K = 0.403 (95% CI 0.098–1.521), *p* <0.0005.

## Discussion

This study found a prevalence of 4.17% of *Trypanosoma* infection which is lower compared to earlier studies in cattle in the same region that established a prevalence of 41% and 29% in Suba and Teso regions of western Kenya, respectively in 2006 (12) and 20.1% in the former Busia district in 2004 (11). This might suggest the impact of improved control strategies against trypanasomiasis, improved local knowledge of the disease or a change in disease dynamics since the other studies were conducted. Between 1999 and 2012 different control strategies were implemented in the region with funding from the European Union for Farming in Tsetse Controlled Areas (FITCA) and Pan African Tsetse And Trypanosomiasis Eradication Council (PATTEC) projects. A study conducted in 2008 demonstrated that 61% of people in western Kenya were aware of tsetse and trypanosome control methods and bush clearing was recently used by almost half of respondents as a control method (19). These interventions might have greatly contributed to the decrease in prevalence of trypansomiasis. However, it is important to determine the current prevalence status as there has been less focus, support and funding for trypanosomiasis in recent years (20) and hence a possibility of an increase in prevalence.

Mapping the positive cases by microscopy (Figure 1A) and by PCR (Figure 1B) showed a similar geographical distribution of trypanasome infection with most cases located in the south western parts of the study area. Spatial mapping of the distribution of *Trypanasoma* species overlaid with the tsetse infested layer showed a number of trypanosomes in the tsetse-free areas which was previously demonstrated in the former Teso and Suba districts (12). Most infections in the tsetse-free areas were of *T. vivax*, which is known to be a mechanically-transmitted parasite by both biting flies and tsetse flies (12, 21). There is a need for further studies to establish whether this is due to mechanical transmission or a change in the distribution of tsetse flies resulting from land use changes due to habitat transformation, degradation and encroachment into the tsetse free areas. The geo-spatial data maps demonstrating the distribution of tsetse flies were developed in the year 1998, using data collected in 1973 (22). There is a likelihood that urbanization and changes in landuse could have modified and even interfered with the tsetse habitats and their distribution.

The risk of trypanasome infection in the study area is similar regardless of the diagnostic test used (Figures 2A,B). The spatially-smoothed risk maps showed the area with the highest risk of trypanasome infection to be the south western region of the study close to Lake Victoria. This may indicate a localized hot-spot for trypanosome transmission in this region which could be targeted for future control initiatives.

Microscopic analysis of trypanosomes is a cheap and simple technique and is easily employed in the field in the detection of trypanaomiasis. It has been shown to have low sensitivity (23). However, in this study microscopy provided an important pre-screening step and enabled comparative assessment of the results obtained. Earlier studies have shown that PCR has a high specificity and sensitivity when diagnosing trypanosome infections (15, 24). Using PCR increased the number of total positive cases identified from 22 to 37 with the diagnosis of *T. brucei* cases being greatly improved as demonstrated in Figure 1B. The predominant *Trypanasoma* infection was *T. vivax* by both tests which agrees with a study conducted in other parts of western Kenya (former Teso and Suba district) (12). There were no positive *T. congolense* cases on PCR and this could be attributed to the primers used which were only specific for *T. congolense* savannah and hence there is a possibility that other sub-species of *T. congolense* could be present but were not detected.

It is important to note that PCR is a very sensitive technique for detecting parasite DNA and not every PCR-positive animal will be clinically ill. This must be considered when relating the prevalence recorded to impacts on animal health and production as these will not be directly proportional. We were not able to make conclusions regarding the clinical status of positive animals in this study. It has been suggested that asymptomatic carrier cattle may impact the success of control programmes targeting the treatment of symptomatic cattle (11).

Human infective *T. b. rhodesiense* infections were not detected even though this part of western Kenya has previously been reported to be a sleeping sickness focus with sporadic cases of HAT (11). Earlier studies reported the prevalence of *T. b. rhodensiense* in cattle to be 21.5% (11). However, the implementation of the PATTEC project in the Lake Victoria region from 2005 to 2012 may have reduced the prevalence of *T. b. rhodesiense* and Kenya has not reported a sleeping sickness case in more than 10 years (25).

## conclusion and Recommendations

This study has demonstrated the crucial role that geospatial data analysis has in improving the understanding of disease status. This will greatly inform future research and policy formulation as it will provide evidence crucial in spearheading informed control strategies to mitigate this disease. The evidence of multiple species co-infections of trypanosomes warrants the need for change of research outlook from single-species to multiple-species investigations to better understand how the different pathogens co-exist in the targeted host. Additionally, it is crucial for continuous studies to be carried out to establish whether cattle in this region are reservoirs for zoonotic trypanosomes which is of great public health importance. This information may help Kenya with its move toward declaring the elimination of HAT as a public health problem.

## Data Availability Statement

All datasets generated for this study are included in the article/Supplementary Material.

## Ethics Statement

The animal study was reviewed and approved by Animal Welfare and Ethical Review Body of the Roslin Institute, University of Edinburgh, UK (AWA004). Written informed consent was obtained from the owners for the participation of their animals in this study.

## Author Contributions

VK and EF: conceptualization. VK and EC: formal analysis. EF: funding acquisition. VK and AK: investigation. EF, EC, and VK: methodology. EF and VK: project administration. EF, PT, and JF: supervision. VK, PT, and EC: writing-original draft. All authors: writing, review, and edit.

## Conflict of Interest

The authors declare that the research was conducted in the absence of any commercial or financial relationships that could be construed as a potential conflict of interest.
